# Clinicopathological Significance of Cancer Stem Cell Markers (OCT-3/4 and SOX-2) in Oral Submucous Fibrosis and Oral Squamous Cell Carcinoma

**DOI:** 10.3390/biomedicines11041040

**Published:** 2023-03-28

**Authors:** Divyambika Catakapatri Venugopal, Cynthia Leslie Caleb, Nandhini Priyadarshini Kirupakaran, Vidyarani Shyamsundar, Soundharya Ravindran, Madhavan Yasasve, Arvind Krishnamurthy, Thamizhchelvan Harikrishnan, Sathasivasubramanian Sankarapandian, Vijayalakshmi Ramshankar

**Affiliations:** 1 Department of Oral Medicine and Radiology, Sri Ramachandra Institute of Higher Education and Research (DU), Porur, Chennai 600116, India; 2 Centre for Oral Cancer Prevention and Research, Sree Balaji Dental College and Hospital, Pallikaranai, Chennai 600100, India; 3 Department of Preventive Oncology (Research), Cancer Institute (WIA), Adyar, Chennai 600020, India; 4 Department of Surgical Oncology, Cancer Institute (WIA), Adyar, Chennai 600020, India; 5 Department of Oral Pathology, Sri Ramachandra Institute of Higher Education and Research (DU), Porur, Chennai 600116, India

**Keywords:** oral submucous fibrosis, immunohistochemistry, malignant transformation, real-time PCR, octamer-binding transcription factor 3/4 (OCT-3/4), sex-determining region Y-box 2 (SOX-2)

## Abstract

Oral submucous fibrosis (OSMF) is highly prevalent in South East Asia with higher rates of malignant transformation in Indian subcontinent. Numerous biomarkers are now being studied to predict disease prognosis and detect malignant alterations at an early stage. Patients with clinically and biopsy-proven oral submucous fibrosis and oral squamous cell carcinoma were included in the study as the experimental group, while patients without a tobacco or betel nut habit who had their third molars surgically removed were included as the healthy control group. For the immunohistochemistry (IHC) investigation, 5-μm slices from formalin-fixed, paraffin-embedded tissue blocks (FFPE) were obtained. Fresh tissues (*n* = 45) from all three groups were collected and gene expression was studied using relative quantitation-based qPCR. The protein expression of octamer-binding transcription factor 3/4 (OCT 3/4) and sex-determining region Y-box 2 (SOX 2) was evaluated in the experimental group and compared with healthy controls. The IHC results showed a significant correlation with the expression of OCT 3/4 (*p* value = 0.000; χ2 = 20.244) and SOX 2 (*p* value = 0.006; χ2 = 10.101) among OSCC and OSMF patients in comparison to healthy controls. Both OCT 3/4 and SOX 2 showed overexpression of four-fold and three-fold in OSMF when compared to OSCC and healthy controls, respectively. This study shows the significant importance of cancer stem cell markers OCT 3/4 and SOX 2 to assess the disease prognosis in OSMF.

## 1. Introduction

Oral submucous fibrosis (OSMF) is a chronic progressive disorder first described by Schwartz in 1952, which is highly prevalent in Southeast Asia. The hallmark of the disease is fibrosis which affects most parts of the oral cavity, pharynx and upper third of the oesophagus and is mainly attributed to arecoline present in the arecanut [1]. OSMF is grouped under oral potentially malignant disorders (OPMD) with malignant transformation rates of 1.5–15% [2]. Numerous theories have been proposed on its pathogenesis and mechanisms of malignant transformation; however, the research on identifying the potential biomarkers for developing targeted therapy is still under progress. Among the various theories proposed, epithelial-to-mesenchymal transition (EMT) plays an important part in fibrosis associated with the disease and its subsequent malignant transformation [3]. It has been hypothesized that hypoxia plays a major contributing factor in malignant transformation, with HIF-1 alpha showing elevated expression at protein and RNA levels [4]. Hypoxic conditions in the tumour microenvironment may also lead to therapeutic resistance by activation of stemness-associated pathways, including octamer-binding transcription factor 3/4 (OCT 3/4) and sex-determining region Y-box 2 (SOX 2) molecular signals [5].

Cancer stem cells (CSCs) are a group of cells that demonstrate therapeutic resistance and are found to aid in tumour progression, recurrence, poor clinical outcomes and overall survival. A significant correlation between the expression of SOX 2 and mucoepidermoid carcinoma was observed when compared to other CSC markers [6]. Systematic review and meta-analysis on gastric cancers showed the association of CSC marker expression to be in correlation with clinicopathological parameters and survival outcome. The authors concluded that CSCs (Gli-1, CD44s, CD44V6, and CD133, including OCT 4) have strong prognostic values and hence could serve as prognostic stratification markers to predict tumour aggressiveness and poor prognosis in gastric cancer patients, thereby helping to identify novel potential therapeutic targets [7]. Stemness- or primitiveness-associated markers have genes involved in the production of transcription factors related to characteristics such as unlimited proliferative potential, self-renewal, and maintenance of pluripotency and have been proposed to play a substantial role in EMT. The master regulators associated with the reprogramming of cells to maintain their undifferentiated state and pluripotency are POU (Pit-Oct-Unc family) 2 contribute domain transcription factor (OCT 3/4), home domain transcription factor (NANOG), and the high mobility group proteins (SOX 2) [8]. These cancer stem cell markers, especially OCT 3/4 and SOX substantially to tumour development and progression, with a significant association of their expression in different grades of OSCC [9]. Apart from the maintenance of pluripotency of embryogenic cells, OCT 3/4 has been shown to play a crucial role in the EMT of stem cells [10]. OCT 3/4 and SOX 2 have been recognized as the tumorigenesis biomarkers of early-stage OSCC, with SOX 2 being suggested as an independent prognostic factor for OSCC [11]. It has been postulated that there is a downregulation of CSC markers due to the atrophied epithelium in OSMF, and a neoplastic transformation would lead to the activation of stemness, leading to the upregulation of these markers in OSCC [12].

Currently, the literature on cancer stem cell markers in assessing the prognosis of oral submucous fibrosis is limited. Expression of CSC markers may guide clinicians in risk stratification of OSMF and anticipating the underlying malignant potential, thereby aiding in the early identification of OSCC. Hence, the current study aims to evaluate and compare the expression of cancer stem cell markers, namely, OCT 3/4 and SOX 2 in OSMF and OSCC using RT-PCR and immunohistochemistry.

## 2. Materials and Methods

### 2.1. Institutional Ethics Approval

The study was carried out at the Outpatient Department (OPD) of Oral Medicine and Radiology, Sri Ramachandra Dental College and Hospital, SRIHER (DU) with Institutional Ethics Committee approval (IEC No. CSP/20/NOV/87/213) dated 8 December 2020 from December 2020 to April 2021.

### 2.2. Sample Collection

The study comprised 120 subjects with clinically and biopsy-proven oral submucous fibrosis (*n* = 47) and oral squamous cell carcinoma patients (*n* = 56) in the experimental group, and patients with no tobacco/betel nut habit, who underwent surgical removal of a third molar were included as the healthy control group (*n* = 17) for two markers (OCT 3/4, SOX 2).

For the OSMF and OSCC groups, demographic details such as age, gender, habits, and duration and frequency of habits were collected. Details of clinical examination, including mouth opening, were recorded for OSMF patients. Patients of the age group 18–70 years of both genders were included as the study samples. The inclusion criteria for the healthy control group (Group A) included healthy controls with no H/O tobacco/betelnut usage. The experimental group consisted of group B and group C, where patients diagnosed clinically and confirmed histologically with OSCC and OSMF respectively were included. Patients with two or more coexisting OPMD, recurrent/second primary OSCC and patients with OSCC (Group B) and OSMF (Group C) who were undergoing treatment/undergone treatment previously were excluded from the study. The clinical diagnostic criteria for OSMF included blanching, palpable fibrotic bands and restricted mouth opening. Mouth opening was measured as interincisal distance using Vernier callipers. Khanna and Andrade classification was employed for the clinical grading of the patients, using functional staging based on mouth opening [13]. The clinical diagnostic criteria for OSCC included ulcer/ulceroproliferative growth anywhere in the oral cavity with indurated margins. Nodal status was assessed, and TNM classification was given. Histological grading of OSCC was performed based on Broders classification as well-differentiated SCC (WDSCC), moderately differentiated SCC (MDSCC), and poorly differentiated SCC (PDSCC) [14].

### 2.3. Immunohistochemistry

Immunohistochemistry was performed by an avidin–biotin method for octamer binding transcription factor (OCT 3/4) and SRY (sex-determining region Y)-box 2 (SOX 2) using 5 µm sections on slides treated with 3-aminopropyltriethoxysilane (APES). Endogenous peroxidase activity was suppressed by soaking the sections in 0.03 percent hydrogen peroxide in distilled water for 10 min, followed by a PBS wash. The sections were pre-incubated with 2% bovine serum albumin (BSA) for 40 min, and any excess was discarded. The sections were pre-incubated with power block (BioGenex) for 10 min and treated with primary antibody EP143 (OCT 4), PathnSitu (pre-diluted) CA, USA and EP103 (SOX 2), PathnSitu (pre-diluted) CA, USA, further incubated overnight at 4 °C in a moisture environment. To identify expression, the Polyexcel HRP/DAB detection system, PathnSitu (Pleasanton, CA, USA), and the BioGenex Super SensitiveTM Detection System (BioGenex, CA, USA) were used. Hematoxylin counterstained slices were dehydrated and mounted in DPX using successive degrees of isopropyl alcohol and xylene. Gene expression was assessed and compared to that of clinically normal oral epithelial cells. The number of positive cells in the epithelium and connective tissue (inflammatory cells) was counted in 10 high-power fields (40×) and the percentage positivity was calculated. To see the small field, counting was performed on a computer monitor using the software ProgRes CapturePro v2.8.8 [15,16,17].

### 2.4. Evaluation of Immunoexpression

The immunohistochemically stained tissue sections were reviewed and scored independently by an oral pathologist, blinded to the clinical parameters. Immunohistochemical scoring for OCT 3/4 was performed as described earlier by research groups [18]. Immunohistochemical scoring for SOX 2 was performed according to the previous published literature [19]. The percentage grade of stained tumour cells greater than 20% was considered as positive immunoexpression of OCT 3/4 and SOX 2.

### 2.5. Homogenization of Tissue Samples

Using a sterile surgical blade, the acquired patient tissue samples (healthy controls = 10; OSMF = 10, OSCC = 25) were cut into tiny pieces. The tissue fragments were placed in separate 2 mL centrifuge tubes with 300 L of TRIzol reagent (Thermo Fischer Scientific Inc., Waltham, MA, USA). Two sterile beads were added to each tube before placing them in a Tissue LyserLT (Qiagen Inc., Venlo, the Netherlands) for 15 min. Following tissue lysis, 700 µL of TRIzol reagent was added to the tubes, which were then resuspended and transferred to new 1.5 mL centrifuge tubes [20].

### 2.6. RNA Isolation from the Tissues

First, 0.2 mL of chloroform was added for every 1 mL of TRIzol reagent, and the tubes were vortexed for 10 to 15 s before incubating at room temperature for 3 min. The samples were centrifuged for 15 min at 4 °C at 12,000 rpm (the mixture was separated into a lower phenol-chloroform phase, interphase, and a colourless upper aqueous phase). RNA was still in the aqueous phase. The aqueous phase was moved to a new tube. Isopropyl alcohol was used to precipitate the RNA (0.5 mL of isopropyl alcohol per 1 mL of TRIzol reagent). The samples were incubated for 10 min at room temperature before being centrifuged at 12,000 rpm for 10 min at 4 °C. The RNA precipitate produced a white pellet on the tube’s side and bottom. The supernatant was removed, and 1 mL of 75% ethanol was added for every 1 mL of TRIzol. The material was vortexed and centrifuged at 7500 rpm for 5 min at 4 °C. The supernatant was discarded, and the RNA pellet was quickly dried. By inverting the tube a few times, the RNA was dissolved in RNase-free water and incubated for 10 min at 55 to 60 °C [21].

### 2.7. cDNA Conversion

The RNA samples were reverse transcribed using the Quantitect Reverse Transcription Kit (Qiagen Inc., Venlo, the Netherlands). A total of 2 g of RNA was required for the cDNA conversion, which included two key steps: genomic DNA removal and reverse transcription. The purified RNA sample was treated with 2 L of gDNA wipe-out buffer for 2 min at 42 °C. The reaction mixture was immediately placed on ice. Following gDNA removal, the RNA sample was ready for reverse transcription with a master mix composed of Quantiscript Reverse Transcriptase, Quantiscript RT Buffer and RT Primer. The reaction was completed at 42 °C and inactivated at 95 °C. The prepared cDNA was kept at 40 °C [22].

### 2.8. Real-Time PCR

The QuantiNova SYBR Green RT-PCR Kit was used for SYBR Green-based real-time amplification (Qiagen Inc., Venlo, the Netherlands). A 20 mL reaction using 10 mL of QuantiNova SYBR Green RT-PCR Master Mix, 1 mL of each forward and reverse primer (Table 1), 6 mL of nuclease-free water, and 2 mL of cDNA was set up. The thermal profile was 30 min at 50 °C, 15 min at 95 °C, 45 cycles of 15 s at 94 °C, 30 s at Tm, and 30 s at 72 °C, followed by a melting curve from 60 °C to 90 °C. All real-time PCRs were performed using a Rotor-Gene Q Real-Time PCR equipment (Qiagen). For gene expression investigations employing OCT 3/4 and SOX 2, triplicate reactions were conducted, and the mean expression value was determined for the following analyses. The (2-ddct) technique was used to compute the relative expression levels of the genes [23].

### 2.9. Statistical Analysis

All statistical analysis was performed in SPSS Version 16 (IBM Corporation, Chicago, IL, USA) [24]. Continuous data (mean + SD) and categorical variables (percentages) were reported. Dunnett’s T3 post hoc test was used to compare clinical characteristics and outcomes between groups. Student’s t-test was used to calculate statistically significant differences between the groups in gene expression (OCT 3/4 and SOX 2) during real-time PCR. The statistical significance was determined using a *p* value less than *p* < 0.05.

## 3. Results

### 3.1. IHC Using Clinical Samples for OCT 3/4

**Figure 1:**
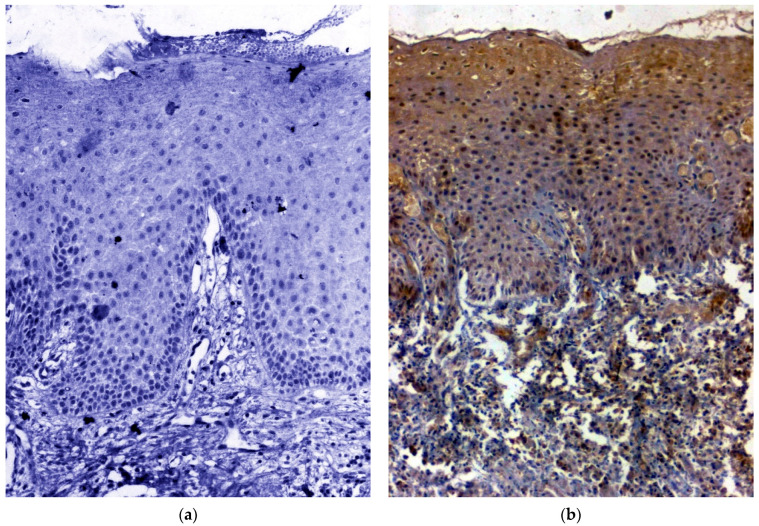

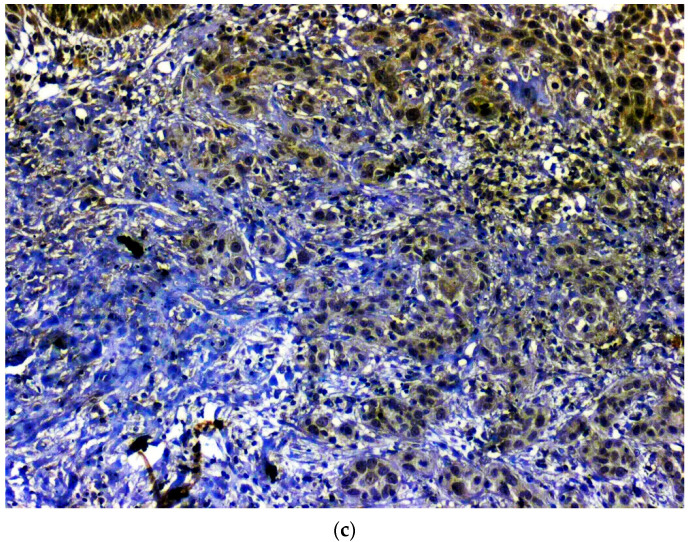
(**a**) IHC for OCT 3/4 in healthy control hyperplastic oral epithelium, showing negative stain, (**b**) IHC for OCT 3/4 in oral submucous fibrosis showing intense nuclear positivity, and (**c**) IHC for OCT 3/4 in MDSCC showing intense nuclear positivity (under 20× magnification).

Out of the 120 samples taken for the IHC, five samples (OSCC: *n* = 2; OSMF: *n* = 3;) were deemed unfit for analysis because of absent or scanty epithelium. Hence, for IHC analysis of OCT 3/4, *n* = 17 in healthy control group, *n* = 54 in OSCC group and *n* = 44 in OSMF group were considered. There was a significant correlation (*p* value = 0.000; χ2 = 20.244) with expression among OSMF and OSCC in comparison with healthy control. Table 2 shows the details of OCT 3/4 expression, which correlated with clinicopathological parameters. We found a significant overexpression (*p* value = 0.001; χ2 = 10.517) with increased age (>47 years), and epithelial abnormality with increased atrophy showing significant overexpression (*p* value = 0.001; χ2 = 18.875). Figure 1a shows the negative immunoexpression of OCT 3/4 in healthy control samples. Figure 1b shows the OCT 3/4 positive immunoexpression in epithelial cells of OSMF samples, with cytoplasmic and nuclear positivity. In OSCC samples, OCT 3/4 demonstrated strong intense nuclear positivity in malignant epithelial cells (Figure 1c). All the microscopic images were taken under 20× magnification.

### 3.2. IHC Using Clinical Samples for SOX 2

**Figure 2:**
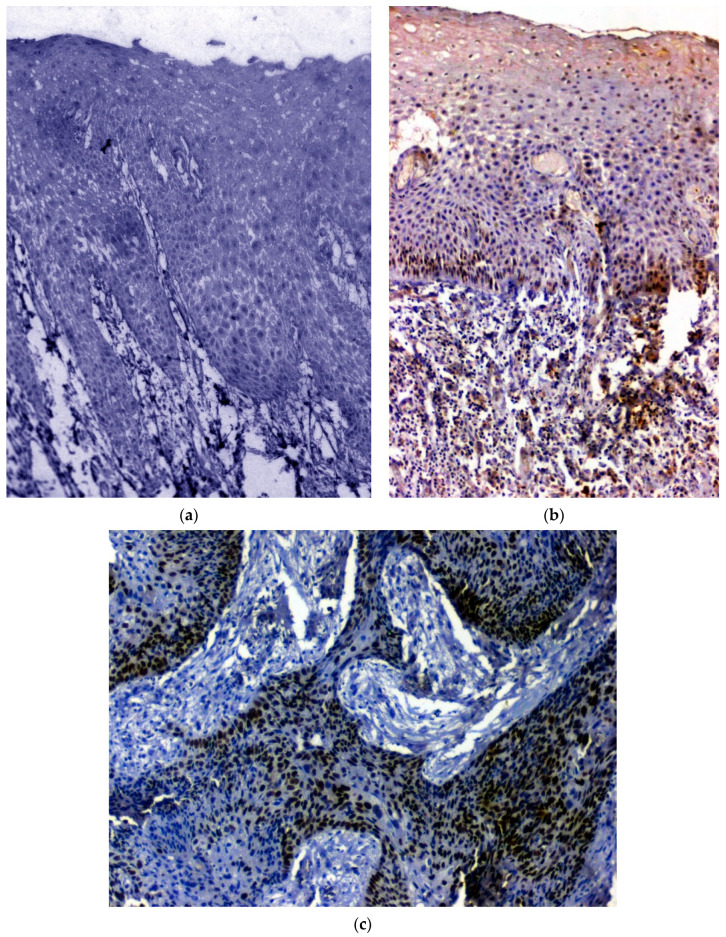
(**a**) IHC for SOX 2 in healthy control hyperplastic oral epithelium, showing negative stain, (**b**) IHC for SOX 2 in oral submucous fibrosis showing intense nuclear positivity, and (**c**) IHC for SOX 2 in MDSCC showing intense nuclear positivity (under 20× magnification).

Out of the 120 samples taken up for the IHC procedure, seven samples (OSCC: *n* = 3; OSMF: *n* = 4) were deemed unfit for analysis because of absent or thin epithelium. Hence, for IHC analysis of SOX 2, *n* = 17 in healthy control group, *n* = 53 in OSCC group and *n* = 43 in OSMF group were considered. There was a significant correlation (*p* value = 0.006; χ2 = 10.101) with SOX 2 expressing higher in OSMF and OSCC patients compared to healthy controls. Table 3 show the details of SOX 2 expression, which correlated with clinicopathological parameters. We found a significant overexpression (*p* value = 0.000; χ2 = 16.911) with increased age (>47 years) and epithelial abnormality with increased atrophy showing significant overexpression (*p* value = 0.027; χ2 = 10.925). Figure 2a shows the negative immunoexpression of SOX 2 in healthy controls. Figure 2b shows the SOX 2 positive immunoexpression in epithelial cells in OSMF, with cytoplasmic and nuclear positivity. In OSCC, SOX 2 demonstrated strong cytoplasmic and nuclear positivity in malignant epithelial cells (Figure 2c). All the microscopic images were taken under 20× magnification.

### 3.3. Real-Time PCR

**Figure 3:**
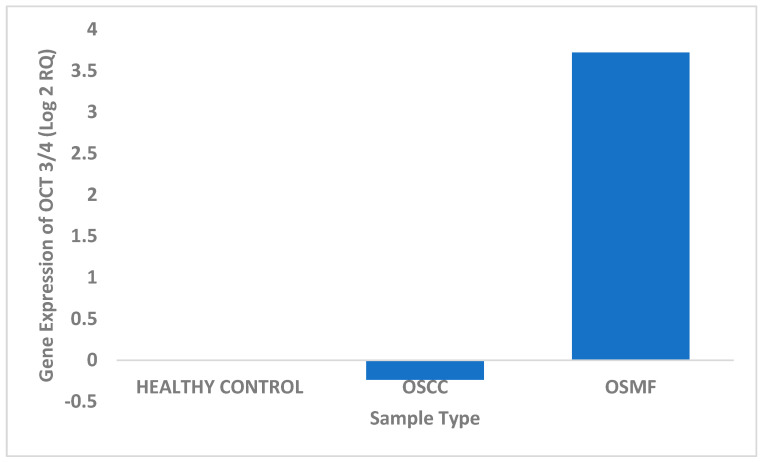
Differential expression of OCT 3/4 in different tissue samples.

The expression of OCT 3/4 was studied in tissue samples using real-time PCR for different patient categories. Based on the obtained results, OCT 3/4 exhibited maximum expression in OSMF tissue samples in comparison to OSCC and healthy controls. We found a significant four-fold upregulation of the OCT 3/4 gene in OSMF samples, and very minimal upregulation in healthy control, with downregulation in OSCC samples (Figure 3 and Table 4). OCT 3/4 expression was found to be statistically significant using Student’s t-test for the studied samples: (i) OSCC in comparison with OSMF with *p* value 0.0017 (<0.05) and (ii) OSMF in comparison with healthy controls with *p* value 0.019 (<0.05).

**Figure 4:**
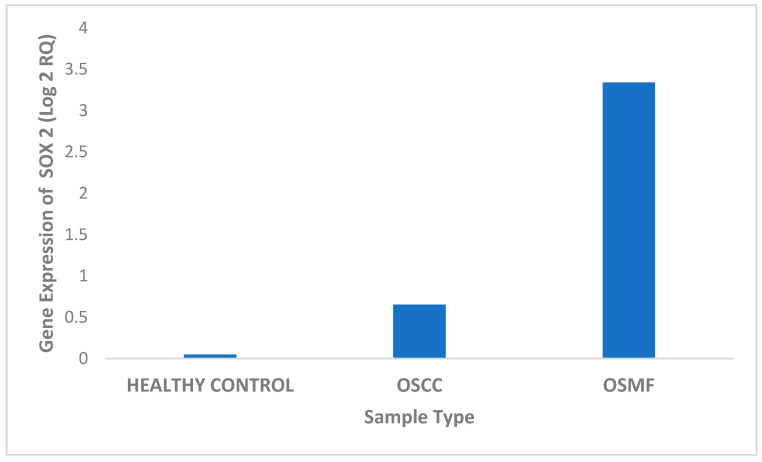
Differential expression of SOX 2 in different tissue samples.

SOX 2 expression was investigated in tissue samples from various patient groups using real-time PCR. According to the findings, SOX 2 expression was highest in OSMF tissue samples compared to OSCC and healthy controls. The SOX 2 gene was shown to be significantly three-fold upregulated in OSMF samples, but very little upregulated in OSCC samples and healthy individuals (Figure 4 and Table 5). Using the Student’s *t*-test, the SOX 2 expression was shown to be statistically significant in the following samples: (i) OSCC in comparison with OSMF with *p* value 0.0055 (0.05) and (ii) OSMF in comparison with healthy controls with *p* value 0.066 (0.05).

## 4. Discussion

The current study evaluates two CSC markers, namely, OCT 3/4 and SOX 2, in OSMF and OSCC in comparison with healthy control patients, using IHC and qPCR. CSCs are implicated in tumour initiation, the aggressive nature of tumours such as chemoresistance, anti-apoptosis, and metastasis and also in tumour persistence and post-treatment recurrence [25,26]. The four transcription factors namely OCT 3/4, SOX 2, Krüppel-like factor 4 (KLF4), and c-MYC are involved in the self-renewal, pluripotency, and reprogramming of stem cells [27]. Among these CSC markers, SOX 2 and OCT 3/4 have been implicated in oral tumorigenesis. SOX 2, located on chromosome 3q26, has been found to play a significant role in embryonic stem cell pluripotency, malignant phenotypes, EMT and has been recognized as an oncogene [28]. OCT 3/4 is involved in the maintenance and production of the undifferentiated state of embryonic stem cells and induced pluripotent stem cells, respectively [27]. OPMD has been found to demonstrate similar molecular genetic traits as seen in OSCC, even in the absence of dysplasia [29]. Hence, it is noteworthy to evaluate the expression of these CSC markers, which are expressed in OSCC, in OPMD to assess their underlying tendency to undergo malignant transformation.

Since the identification of OSMF in arecanut chewers, numerous mechanisms have been proposed regarding its potential to undergo malignant transformation [1]. However, the initiation of tumour development in the pre-existing atrophic epithelium in OSMF is ambiguous and could be attributed to the accumulation of CSC markers, providing the required potential for transformation to malignancy. The increased expression of CSC markers, namely SOX 2 and Bmi-1, in a retrospective cohort study conducted on OSMF observed the role of the abovementioned CSC markers in abnormal proliferation associated with OSCC transformation [12]. OCT 3/4 is involved in various oncogenic processes such as initiation, progression and metastasis of different oral cancers, including OSCC [30]. The expression of SOX 2 and OCT 3/4 has been extensively studied in OSCC [31], and they have been found to be significantly expressed. Hence, the authors have suggested SOX 2 and OCT 3/4 to be prognostic markers indicating disease survival, including chemoresistance in OSCC.

Although numerous studies have shown increased expression of OCT 3/4 in OSCC, studies on OCT 3/4 and SOX 2 in OSMF are limited, especially about the hypothesis of rebound activity of stem cell markers in the atrophic epithelium in OSMF leading to dysplasia and further progression to invasive malignancy [12]. It has been suggested that wild-type p53 (Wt-p53) was found to be highly expressed in the atrophic epithelium in OSMF, with suppression of stemness by downregulating the CSC markers such as cMYC, OCT 3/4 and SOX 2. However, the expression of mutant p53 and upregulation of cMYC causes an alteration in stemness, thereby leading to clonal expansion, invasion and further progression to OSCC [32,33,34] Study conducted by Abeyasinghe et al. 2021 assessing the role of CSC marker Bmi—1 expression in OSMF and malignancy arising from OSMF, showed significantly higher expression of Bmi-1 in OSCC arising from OSMF [35]. A similar study evaluating the immunoexpression of OCT 3/4 and SOX 2 in OSMF showed significantly increased expression in OSMF compared to healthy controls; however, no significant difference concerning grading was noted, which is in accordance with the present study [18]. A clinical cohort study evaluating the immunoexpression of Ki67, Bmi-1 and SOX 2 in OSMF showed high expression of these markers in OSMF tissues with dysplasia, thus postulating these CSC markers to be the triggers towards carcinogenesis in OSMF [36].

CSC markers apart from SOX 2, OCT 3/4, such as CD44, CD133, glucose-regulated protein 78 (Grp-78), Grp-96, and NANOG were upregulated upon areca nut exposure and correlate with worse prognosis in areca nut-induced OSCC [12]. The results of the present study, with increased immunoexpression of SOX 2 observed in OSMF and OSCC compared to healthy controls, follow the study conducted by Xie et al. 2020 [36]. The current study, showing increased expression of OCT 3/4 and SOX 2 in OSMF, suggests these markers to play a vital role in the transformation of the atrophic epithelium in OSMF into dysplasia and eventually into OSCC. The RT-PCR results of the present study showed significant upregulation of these CSC markers (OCT 3/4 and SOX 2) in OSMF compared to healthy controls and OSCC. The study conducted by Habu and team in 2015 showed increased expression of CSC markers in the side population of tongue cancers compared to the main population, with these markers thus playing an important role in invasiveness and cell motility [30]. A study evaluating SOX 2 immunoexpression in epithelial dysplasia and OSCC found 7% of cases with dysplasia to show positive immunoexpression as compared to 36% of OSCC. However, the increased expression of SOX 2 was observed in the early stages of tumours and N0 cases when compared to advanced stages. The structural and functional transformation of normal cells to precancerous cells, followed by dysplasia and finally to OSCC, is a multivariate process. Precancerous cancer stem cell (pCSC) markers have been associated with OPMD and CSC in OSCC. It has been postulated that the pCSC plays a vital role in the initiation and development of OCCC from precancerous cells to OSCC, with attributes from the local microenvironment and epigenetic factors. The identification of pCSCs in OPMD can aid in evaluating the risk of malignant transformation and identify the disease progression in the early stages [37].

The limitation of the current study was the smaller sample size of healthy controls, but in the current study, these CSC markers were found to be not expressed in 88% of the healthy controls. However, with these preliminary results obtained, future studies can be planned with other CSC markers, larger sample size in different clinical stages of OSMF to assess the disease progression. The present study showed, that the higher expression of OCT 3/4 and SOX 2 in OSMF could be a valuable attribute in predicting the disease prognosis in OSMF. Future studies can be aimed to evaluate the role of other CSC markers in OSMF to develop a CSC biomarker panel to improve the predictive ability in assessing the prognosis and its associated malignant transformation.

## 5. Conclusions

The current study has evaluated the quantitative expression of CSC markers, namely, OCT 3/4 and SOX 2 using IHC and RT-PCR in OSMF and OSCC. The present study showed significant expression of OCT 3/4 and SOX 2 in OSMF and OSCC compared to healthy controls. Hence, these CSC markers could serve as adjuncts to the clinician in identifying the high-risk cases which can undergo carcinomatous transformation. Future research will aim for the evaluation the expression of different CSCs in various clinical stages of OSMF for a better understanding of disease progression.

## Figures and Tables

**Table 1:** OCT 3/4 and SOX 2 primer sequences.

Gene Name	Forward Sequence	Reverse Sequence
OCT 3/4	5′-GACAGGGGGAGGGGAGGAGCTAGG-3′	5′-CTTCCCTCCAACCAGTTGCCCCAAAC-3′
SOX 2	5′-GGGAAATGGGAGGGGTGCAAAAGAGG-3′	5′-TTGCGTGAGTGTGGATGGGATTGGTG-3′

**Table 2:** Clinicopathological features of OCT 3/4 expression in OSMF, OSCC and healthy controls.

Criteria	Total (*n* = 115)	OCT ¾ Negative (*n* = 45)	OCT ¾ Positive (*n* = 70)
**Gender**			
Male	83	32 (36.8%)	51 (58.6%)
Female	32	13 (39.4%)	19 (57.6%)
**Age**			
>47 years	60	15 (25%)	45 (75%)
<47 years	55	30 (54.5%)	25 (45.5%)
***p*** **value = 0.001; χ2 = 10.517**			
**Diagnosis**			
Healthy control	17	15 (88.2%)	2 (11.8%)
OSMF	44	14 (31.8%)	30 (68.2%)
OSCC	54	16 (29.6%)	38 (70.4%)
***p*** **value = 0.000; χ2 = 20.244**			
**Epithelial Nature**			
Healthy control	9	9(100%)	0 (0%)
Hypertrophic	16	8 (50%)	8 (50%)
Atrophic	33	12(36.4%)	21 (63.6%)
Dysplasia	3	0 (0%)	3 (100%)
OSCC	54	16 (29.6%)	38 (70.4%)
***p*** **value = 0.001; χ2 = 18.875**			
**Habits (OSMF *n* = 44)**			
Pan	27	9 (33.3%)	18 (66.7%)
Betel Nut	6	3 (50%)	3 (50%)
Maava	9	2 (22.2%)	7 (77.8%)
Gutka	2	0 (0%)	2 (100%)
**Inflammation (OSMF *n* = 44)**			
Mild	15	5 (33.3%)	10 (66.7%)
Moderate	15	4 (26.7%)	11 (73.3%)
Severe	14	5 (35.7%)	9 (64.3%)
**Fibrosis (OSMF *n* = 44)**			
Mild	10	1 (10%)	9 (90%)
Moderate	17	6 (35.3%)	11 (64.7%)
Severe	17	7 (41.2%)	10 (58.8%)
**Vascularity (OSMF *n* = 44)**			
Healthy Control	7	1 (14.3%)	6 (85.7%)
Reduced	22	8 (36.4%)	14 (63.6%)
Enlarged and increased	15	5 (33.3%)	10(66.7%)
**OSMF Clinical Stage (OSMF *n* = 44)**			
Stage I	8	3 (37.5%)	5 (62.5%)
Stage II	18	8 (44.4%)	10 (55.6%)
Stage III	15	2 (13.3%)	13 (86.7%)
Stage IV	3	1(33.3%)	2(66.7%)
**OSCC Histological Stage (OSCC *n* = 54)**			
WDSCC	27	9 (33.3%)	18 (66.7%)
MDSCC	24	5 (20.8%)	19 (79.2%)
PDSCC	3	2 (66.7%)	1 (33.3%)
**OSCC Clinical Stage (OSCC *n* = 54)**			
Stage I	8	3 (37.5%)	5 (62.5%)
Stage II	16	5 (31.2%)	11 (68.8%)
Stage III	15	6 (40.0%)	9 (60.0%)
Stage IV	15	2 (13.3%)	13 (86.7%)

**Table 3:** Clinicopathological features of SOX 2 expression in OSMF, OSCC and healthy controls.

Criteria	Total (*n* = 113)	SOX 2 Negative (*n* = 69)	SOX 2 Positive (*n* = 44)
**Gender**			
Male	81	49 (60.5%)	32 (39.5%)
Female	32	20 (62.5%)	12 (37.5%)
**Age**			
>47 years	60	26 (43.3%)	34 (56.7%)
<47 years	53	43 (81.1%)	10 (18.9%)
***p*** **value = 0.000; χ2 = 16.911**			
**Diagnosis**			
Healthy Control	17	16 (94.1%)	1 (5.9%)
OSMF	43	26 (60.5%)	17 (39.5%)
OSCC	53	27 (50.9%)	26 (49.1%)
***p*** **value = 0.006; χ2 = 10.101**			
**Epithelial Nature**			
Healthy Control	9	8 (88.9%)	1 (11.1%)
Hypertrophic	16	27 (87.5%)	2 (12.5%)
Atrophic	32	19(59.4%)	13 (40.6%)
Dysplasia	3	1 (33.3%)	2 (66.7%)
OSCC	53	27 (50.9%)	26 (49.1%)
***p*** **value = 0.027; χ2 = 10.925**			
**Habits (OSMF *n* = 43)**			
Pan	27	19 (70.4%)	8 (29.6%)
Betel Nut	6	3 (50.0%)	3 (50.0%)
Maava	9	4 (44.4%)	5 (55.6%)
Gutka	1	0 (0%)	1 (100%)
**Inflammation (OSMF *n* = 43)**			
Mild	14	8 (57.1%)	6 (42.9%)
Moderate	15	10 (66.7%)	5 (33.3%)
Severe	14	8 (57.1%)	6 (42.9%)
**Fibrosis (OSMF *n* = 43)**			
Mild	9	6 (66.7%)	3 (33.3%)
Moderate	17	11 (64.7%)	6 (35.3%)
Severe	17	9 (52.9%)	8 (47.1%)
**Vascularity (OSMF *n* = 43)**			
Healthy Control	6	4 (66.7%)	2 (33.3%)
Reduced	22	13 (59.1%)	9 (40.9%)
Enlarged and increased	15	9 (60.0%)	6(40.0%)
**OSMF Clinical Stage (OSMF *n* = 43)**			
Stage I	7	3 (42.9%)	4 (57.1%)
Stage II	18	14 (77.8%)	4 (22.2%)
Stage III	15	8 (53.3%)	7 (46.7%)
Stage IV	3	1(33.3%)	2(66.7%)
**OSCC Histological Stage (OSCC *n* = 53)**			
WDSCC	27	15 (55.6%)	12 (44.4%)
MDSCC	24	10 (41.7%)	14 (58.3%)
PDSCC	2	2 (100%)	0 (0%)
**OSCC Clinical Stage (OSCC *n* = 53)**			
Stage I	8	5 (62.5%)	3 (37.5%)
Stage II	15	5 (33.3%)	10 (66.7%)
Stage III	15	7 (46.7%)	8 (53.3%)
Stage IV	15	10 (66.7%)	5 (33.3%)

**Table 4:** Expression levels of OCT 3/4 in OSCC, OSMF and healthy control samples.

Patient Sample Type	Log 2 RQ
Healthy Control	0.004444
OSCC	−0.2368
OSMF	3.721

**Table 5:** Expression levels of SOX 2 in OSCC, OSMF and healthy control samples.

Patient Sample Type	Log 2 RQ
Healthy Control	1.18 × 10^−15^
OSCC	0.6524
OSMF	3.34

## Data Availability

The data associated with the manuscript are available from the first and corresponding authors.

## References

[1] Ekanayaka R.P., Tilakaratne W.M. (2016). Oral Submucous Fibrosis: Review on Mechanisms of Malignant Transformation. Oral Surg. Oral Med. Oral Pathol. Oral Radiol..

[2] Wang Y.-Y., Tail Y.-H., Wang W.-C., Chen C.-Y., Kao Y.-H., Chen Y.-K., Chen C.-H. (2014). Malignant Transformation in 5071 Southern Taiwanese Patients with Potentially Malignant Oral Mucosal Disorders. BMC Oral Health.

[3] Hu Y., Jian X., Peng J., Jiang X., Li N., Zhou S. (2008). Gene Expression Profiling of Oral Submucous Fibrosis Using Oligonucleotide Microarray. Oncol. Rep..

[4] Tilakaratne W.M., Iqbal Z., Teh M.T., Ariyawardana A., Pitiyage G., Cruchley A., Stewart J.E., Hagi-Pavli E., Lalli A., Waseem A. (2008). Upregulation of HIF-1alpha in Malignant Transformation of Oral Submucous Fibrosis. J. Oral Pathol. Med..

[5] Li Y.-C., Cheng A.-J., Lee L.-Y., Huang Y.-C., Chang J.T.-C. (2019). Multifaceted Mechanisms of Areca Nuts in Oral Carcinogenesis: The Molecular Pathology from Precancerous Condition to Malignant Transformation. J. Cancer.

[6] Sadeghi H., Saffar H., Taheri P., Yazdani F., Etebarian A. (2022). Prognostic Significance of Cancer Stem Cell Markers in Patients With Salivary Gland Carcinomas. Appl. Immunohistochem. Mol. Morphol..

[7] Razmi M., Ghods R., Vafaei S., Sahlolbei M., Zanjani L.S., Madjd Z. (2021). Clinical and Prognostic Significances of Cancer Stem Cell Markers in Gastric Cancer Patients: A Systematic Review and Meta-Analysis. Cancer Cell Int..

[8] Major A.G., Pitty L.P., Farah C.S. (2013). Cancer Stem Cell Markers in Head and Neck Squamous Cell Carcinoma. Stem Cells Int..

[9] Vaiphei K., Sinha S.K., Kochhar R. (2014). Comparative Analysis of Oct4 in Different Histological Subtypes of Esophageal Squamous Cell Carcinomas in Different Clinical Conditions. Asia Pac. J. Cancer Prev..

[10] Tsai L.-L., Hu F.-W., Lee S.-S., Yu C.-H., Yu C.-C., Chang Y.-C. (2014). Oct4 Mediates Tumor Initiating Properties in Oral Squamous Cell Carcinomas through the Regulation of Epithelial-Mesenchymal Transition. PLoS ONE.

[11] Fu T.-Y., Hsieh I.-C., Cheng J.-T., Tsai M.-H., Hou Y.-Y., Lee J.-H., Liou H.-H., Huang S.-F., Chen H.-C., Yen L.-M. (2016). Association of OCT4, SOX2, and NANOG Expression with Oral Squamous Cell Carcinoma Progression. J. Oral Pathol. Med..

[12] Sharma M., Fonseca F.P., Hunter K.D., Radhakrishnan R. (2020). Loss of Oral Mucosal Stem Cell Markers in Oral Submucous Fibrosis and Their Reactivation in Malignant Transformation. Int. J. Oral. Sci..

[13] Khanna J.N., Andrade N.N. (1995). Oral Submucous Fibrosis: A New Concept in Surgical Management. Report of 100 Cases. Int. J. Oral Maxillofac. Surg..

[14] Akhter M., Hossain S., Rahman Q.B., Molla M.R. (2011). A Study on Histological Grading of Oral Squamous Cell Carcinoma and Its Co-Relationship with Regional Metastasis. J. Oral Maxillofac. Pathol..

[15] Raungrut P., Petjaroen P., Geater S.L., Keeratichananont W., Phukaoloun M., Suwiwat S., Thongsuksai P. (2017). Methylation of 14-3-3σ Gene and Prognostic Significance of 14-3-3σ Expression in Non-Small Cell Lung Cancer. Oncol. Lett..

[16] Tavassol F., Starke O.F., Kokemüller H., Wegener G., Müller-Tavassol C.C.M., Gellrich N.-C., Eckardt A. (2011). Prognostic Significance of Heat Shock Protein 70 (HSP70) in Patients with Oral Cancer. Head Neck Oncol..

[17] Venugopal D.C., Ravindran S., Shyamsundar V., Sankarapandian S., Krishnamurthy A., Sivagnanam A., Madhavan Y., Ramshankar V. (2022). Integrated Proteomics Based on 2D Gel Electrophoresis and Mass Spectrometry with Validations: Identification of a Biomarker Compendium for Oral Submucous Fibrosis—An Indian Study. J. Pers. Med..

[18] Vidhale R.G., Pereira T., Lalai M.N., Anjali A.K., Jain A., Pereira C. (2022). Qualitative Expression of Sox2 and Oct4 in Oral Submucous Fibrosis: An Immunohistochemical Study. J. Pharm. Res. Int..

[19] Ren Z.-H., Zhang C.-P., Ji T. (2016). Expression of SOX2 in Oral Squamous Cell Carcinoma and the Association with Lymph Node Metastasis. Oncol. Lett..

[20] Smith B., Li N., Andersen A.S., Slotved H.C., Krogfelt K.A. (2011). Optimising Bacterial DNA Extraction from Faecal Samples: Comparison of Three Methods. Open Microbiol. J..

[21] RNA Extraction. https://www.usbio.net/protocols/rna-extraction.

[22] Picard-Meyer E., Peytavin de Garam C., Schereffer J.L., Marchal C., Robardet E., Cliquet F. (2015). Cross-Platform Evaluation of Commercial Real-Time SYBR Green RT-PCR Kits for Sensitive and Rapid Detection of European Bat Lyssavirus Type 1. BioMed Res. Int..

[23] Yip C.C.-Y., Ho C.-C., Chan J.F.-W., To K.K.-W., Chan H.S.-Y., Wong S.C.-Y., Leung K.-H., Fung A.Y.-F., Ng A.C.-K., Zou Z. (2020). Development of a Novel, Genome Subtraction-Derived, SARS-CoV-2-Specific COVID-19-Nsp2 Real-Time RT-PCR Assay and Its Evaluation Using Clinical Specimens. Int. J. Mol. Sci..

[24] How to Cite IBM SPSS Statistics or Earlier Versions of SPSS. https://www.ibm.com/support/pages/how-cite-ibm-spss-statistics-or-earlier-versions-spss.

[25] Chen C., Méndez E., Houck J., Fan W., Lohavanichbutr P., Doody D., Yueh B., Futran N.D., Upton M., Farwell D.G. (2008). Gene Expression Profiling Identifies Genes Predictive of Oral Squamous Cell Carcinoma. Cancer Epidemiol. Biomark. Prev..

[26] Zhou S., Zhu Y., He Z., Zhang D., Guo F., Jian X., Zhang C. (2019). Long Non-Coding RNA Expression Profile Associated with Malignant Progression of Oral Submucous Fibrosis. J. Oncol..

[27] Baek K.-H., Choi J., Pei C.-Z. (2020). Cellular Functions of OCT-3/4 Regulated by Ubiquitination in Proliferating Cells. Cancers.

[28] Liu K., Xie F., Zhao T., Zhang R., Gao A., Chen Y., Li H., Zhang S., Xiao Z., Li J. (2020). Targeting SOX2 Protein with Peptide Aptamers for Therapeutic Gains against Esophageal Squamous Cell Carcinoma. Mol. Ther..

[29] Mithani S.K., Mydlarz W.K., Grumbine F.L., Smith I.M., Califano J.A. (2007). Molecular Genetics of Premalignant Oral Lesions. Oral Dis..

[30] Habu N., Imanishi Y., Kameyama K., Shimoda M., Tokumaru Y., Sakamoto K., Fujii R., Shigetomi S., Otsuka K., Sato Y. (2015). Expression of Oct3/4 and Nanog in the Head and Neck Squamous Carcinoma Cells and Its Clinical Implications for Delayed Neck Metastasis in Stage I/II Oral Tongue Squamous Cell Carcinoma. BMC Cancer.

[31] de Vicente J.C., Donate-Pérez Del Molino P., Rodrigo J.P., Allonca E., Hermida-Prado F., Granda-Díaz R., Rodríguez Santamarta T., García-Pedrero J.M. (2019). SOX2 Expression Is an Independent Predictor of Oral Cancer Progression. J. Clin. Med..

[32] Anura A., Kazi A., Pal M., Paul R.R., Sengupta S., Chatterjee J. (2018). Endorsing Cellular Competitiveness in Aberrant Epithelium of Oral Submucous Fibrosis Progression: Neighbourhood Analysis of Immunohistochemical Attributes. Histochem. Cell Biol..

[33] Sachdeva M., Zhu S., Wu F., Wu H., Walia V., Kumar S., Elble R., Watabe K., Mo Y.-Y. (2009). P53 Represses C-Myc through Induction of the Tumor Suppressor MiR-145. Proc. Natl. Acad. Sci. USA.

[34] Wang T.Y., Peng C.-Y., Lee S.-S., Chou M.-Y., Yu C.-C., Chang Y.-C. (2016). Acquisition Cancer Stemness, Mesenchymal Transdifferentiation, and Chemoresistance Properties by Chronic Exposure of Oral Epithelial Cells to Arecoline. Oncotarget.

[35] Abeyasinghe W., Tennakoon P., Jayasooriya P. (2021). Evaluation of cancer stem cell marker, BMI-1 expression in oral submucous fibrosis with and without malignant transformation. Oral Surg. Oral Med. Oral Pathol. Oral Radiol..

[36] Xie C., Feng H., Zhong L., Shi Y., Wei Z., Hua Y., Ji N., Li J., Tang Z., Chen Q. (2020). Proliferative Ability and Accumulation of Cancer Stem Cells in Oral Submucous Fibrosis Epithelium. Oral Dis..

[37] Čēma I., Dzudzilo M., Kleina R., Franckevica I., Svirskis Š. (2021). Correlation of Soluble CD44 Expression in Saliva and CD44 Protein in Oral Leukoplakia Tissues. Cancers.

